# Healthcare prioritisation and inequitable inequalities: why a child health perspective should be incorporated into the current NHS guidance

**DOI:** 10.1136/archdischild-2023-325634

**Published:** 2023-05-19

**Authors:** Sapfo Lignou, Ingrid Wolfe

**Affiliations:** 1 Ethox Centre and Wellcome Centre for Ethics and Humanities, University of Oxford, Oxford, UK; 2 Guy’s and St Thomas’ NHS Foundation Trust, London, UK; 3 Institute of Women and Children's Health, King's College London, London, UK

**Keywords:** Child Health, Covid-19, Ethics, Healthcare Disparities, Paediatrics

One of the main aims of the post-COVID National Health System (NHS) is to tackle inequalities in experience, access and health outcomes that compromised the health of the most vulnerable patients in the time of crisis.^1^ This aim suggests that in reducing the current care backlog for treatment, equity considerations should be traded off against efficiency when prioritising healthcare. Giving priority to categories of care or population groups is necessary to address preventable and undesirable health inequalities in keeping with Marmot’s proportionate universalist approach to reducing inequalities in health,^2^ and reflects our moral intuitions to ensure that those who have already experienced significant misfortune are not further disadvantaged.

Equity considerations regarding healthcare prioritisation are currently framed by socioeconomic and ethnic health disparities. Supported by evidence^3^ showing the disproportionate impact of the pandemic on the health of ethnic minority and lower socioeconomic groups, these considerations are important in current decision-making processes. Nevertheless, they do not capture the magnitude of health inequalities that should be considered and addressed as part of a postpandemic project to address issues of health justice.

To address health and care inequalities efficiently and fairly, the stage of a patient’s life must be included in priority setting. For the paediatric population this is particularly important. Adult ill health can begin in early childhood. Consider, for instance, the impact of adverse exposures on neurodevelopmental system). Furthermore, the combination of being a child and being in a socially disadvantaged group is likely further to exacerbate vulnerability. This is particularly evident in the case of children with chronic and complex conditions, who, due to poor care coordination and system failures, already faced poorer health outcomes prior to the pandemic compared to their counterparts in many other European countries. Disruptions to planned outpatient care have further exposed these children to further risk and avoidable harm.^4^ Unmet needs are closely associated with disadvantage, raising profound ethical and policy concerns. In the case of children these concerns are *distinctly important* as not only the immediate interests of a child are affected but also their developmental and future interests.

Children who experience multiple interacting and often compounding disadvantages are more likely to experience diminished health and quality of life and reduced opportunities to experience a range of other important goods,^5^ such as attending school, socialising with friends and developing skills and abilities necessary to maximise developmental potential and pursue life goals. Given the cumulative effects on long-term life prospects, health and care inequalities in childhood may translate into social and economic inequalities over time. Worryingly, many children who experienced missed opportunities to receive care crucial for their development during the peak of the pandemic may continue to miss care in the current stages of health system turmoil and economic constraints because of prioritisation processes that are not sensitive to their specific needs.

If we believe that children’s life course stage must be taken into account when healthcare prioritisation decisions are made, then we must devise strategies to equalise their health prospects and increase their care opportunities. How might this work? Transferring resources from the care of adult patients to children has been argued as an ethically justifiable policy to address the problem of scarce healthcare resources in the ethics literature.^6^ As it is the opportunity to benefit, rather than a discriminatory assessment of children’s immediate social or economic worth, that matters in this argument, this view seems intuitively appealing. Children’s healthcare should be prioritised to avoid the distinctively bad and unfair outcomes that happen without an explicit means of giving visibility to their longer term capacity to benefit. Given that all people age, securing childhood as a more important stage has also been suggested as prudent resource allocation strategy that most people would reasonably accept.

Yet despite its appeal, this approach presents several challenges. Having to choose between candidates for treatment who differ in many respects requires decisions about how other disadvantages within and across population groups should feature. Furthermore, we must consider what sacrifices we are willing to make in order to redress injustices on children. For instance, deprioritising adult care may require favouring equity over the more usual efficiency maxim that underpins resource allocation decisions. Addressing health inequities requires difficult choices informed by reflection on values which are ‘essentially contestable’ and lead to very different strategies.

The complexity of such decisions, however, does not discharge us from our duty, as society, to be just to children. A pragmatic, alternative approach that requires that children’s disadvantages do not add to current health injustices is needed. Currently, the omission of an ‘age-related’ needs criterion as a distinguishing characteristic among patients currently competing for treatment in NHS healthcare prioritisation decisions obscures issues of health justice particularly in the case of children, in the post-COVID era. Introducing an ‘age-related needs’ criterion would serve as a crude measure of the priority given to care, taking into account the specific needs of patients at different stages of life. This criterion would help identify structural care inequalities and health adversity between different age cohorts that must be addressed. In addition to using measures of healthy life expectancy such as quality-adjusted life years (QALYs) or disability-adjusted life years (DALYs) that take into account the remaining years of life, it is important to consider child-specific health factors as part of a comprehensive understanding of age-related needs. This approach would enable decision-making that is aligned with and inclusive of the unique care needs of the pediatric population, such as timely identification of health deterioration or identification of missed developmental milestones. By incorporating an 'age-related' needs criterion, we can extend the concept of a‘fair chance of treatment’ for children, ensuring that their actual and developmental well-being are properly considered in healthcare prioritisation plans without, however, adding further disadvantages to other groups by arbitrary restricting their access to care.

There are important ethical reasons for which health inequities should be at the centre of healthcare policy concerns; in particular for children. Preventable and undesirable health and care inequalities in childhood reduce the well-being of those disadvantaged, and may have larger implications for human, social and economic development in our societies.^7^ As such, addressing health equity considerations is important for decisions about elective care prioritisation, and for the imminent consequences on the health system and the future challenges they may bring.

Ethical questions must be considered in policy design, balancing short-term efficiency with a vision of a just society. There is a compelling case for addressing unfair health and care inequalities within and across different generations currently served by the NHS.
